# Support for Natural Small-Molecule Phenols as Anxiolytics

**DOI:** 10.3390/molecules22122138

**Published:** 2017-12-06

**Authors:** Xiaohong Wang, Yahong Chen, Qiang Wang, Lu Sun, Guiyun Li, Chanxi Zhang, Jianmei Huang, Lei Chen, Haifeng Zhai

**Affiliations:** 1 School of Chinese Materia Medica, Beijing University of Chinese Medicine, Liangxiang Town, Fangshan District, Beijing 102488, China; wangxiaohong2698@163.com (X.W.); chenyahong@bucm.edu.cn (Y.C.); 18811020296@163.com (Q.W.); sunlu_2013@163.com (L.S.); lgy198806@163.com (G.L.); xxdpgy@foxmail.com (C.Z.); 2 National Institute on Drug Dependence, Peking University, 38#, Xueyuan Road, Haidian District, Beijing 100191, China; leichen@bjmu.edu.cn

**Keywords:** anxiety, phenolic hydroxyl group, elevated plus-maze test, phenolic compounds, neuronal spike, hippocampus

## Abstract

Natural small-molecule phenols (NSMPs) share some bioactivities. The anxiolytic activity of NSMPs is attracting attention in the scientific community. This paper provides data supporting the hypothesis that NSMPs are generally anxiolytic. The anxiolytic activities of seven simple phenols, including phloroglucinol, eugenol, protocatechuic aldehyde, vanillin, thymol, ferulic acid, and caffeic acid, were assayed with the elevated plus maze (EPM) test in mice. The oral doses were 5, 10 and 20 mg/kg, except for phloroglucinol for which the doses were 2.5, 5 and 10 mg/kg. All tested phenols had anxiolytic activity in mice. The phenolic hydroxyl group in 4-hydroxycinnamic acid (4-OH CA) was essential for the anxiolytic activity in the EPM test in mice and rats compared to 4-chlorocinnamic acid (4-Cl CA). The in vivo spike recording of rats’ hippocampal neurons also showed significant differences between 4-OH CA and 4-Cl CA. Behavioral and neuronal spike recording results converged to indicate the hippocampal CA1 region might be a part of the anxiolytic pathways of 4-OH CA. Therefore, our study provides further experimental data supporting NSMPs sharing anxiolytic activity, which may have general implications for phytotherapy because small phenols occur extensively in herbal medicines.

## 1. Introduction

Small-molecule phenols are extensively found in natural sources, and share some pharmacological properties, such as anti-oxidant [1], anti-microorganism [2], muscle-relaxant [3], and neuro-protective [4] properties. Accumulating reports have been published on the anxiolytic activities of natural small-molecule phenols (NSMPs), including, but not limited to, honokiol [5], paeonol [6], quercetin [7], carvacrol [8], kaempferol [9], and danshensu [10]. Our research group is focusing on anxiolytic natural products, and some anxiolytic natural phenols were found, including juncusol [11,12], effusol [13], and orcinol [14]. Initial reviews of the literature and our findings triggered us to consider other NSMPs and their mechanism of action. In the present study, we demonstrated that phloroglucinol, eugenol, protocatechuic aldehyde, vanillin, thymol, ferulic acid, and caffeic acid are anxiolytic. To verify the key role of phenolic hydroxyl (OH) in NSMPs, the anxiolytic effect of 4-hydroxycinnamic acid (4-OH CA) and 4-chlorocinnamic acid (4-Cl CA) were compared in mice and rats with behavioral tests, and the neuroactive differences between 4-OH CA and 4-Cl CA were revealed by testing their effects on neuronal spikes in the hippocampus of rats. Based on a deep analysis of the literature and our findings, we partially validated the hypothesis that the anxiolytic activity is a common property of NSMPs, and the general implications for phytotherapy are discussed.

## 2. Materials and Methods

### 2.1. Animals

Male CD-1 mice (26–28 g) and male Sprague-Dawley rats (260–280 g) were purchased from Beijing Vital River Laboratory Animal Technology Ltd. (Beijing, China). Animals were housed at a controlled temperature (22 ± 2 °C) in a 12-h light/12-h dark cycle with 50–60% relative humidity for seven days before the behavioral tests. Tap water and a standard solid diet were freely available. All manipulations on animals were in accordance with the National Institutes of Health Guide for the Care and Use of Laboratory Animals, with the approval of the Committee of Biomedical Ethics of Peking University Health Science Center (LA2012-034).

### 2.2. Drugs

The phenols tested in this study were all commercially available: protocatechuic aldehyde (purity 98.2%), ferulic acid (purity 99.6%), and caffeic acid (purity 98%) were purchased from the National Institute for the Control of Pharmaceutical and Biological Products (Beijing, China); 2-hydroxybenzyl alcohol (purity 98%), vanillin (purity 99%), eugenol (purity 99%), and thymol (purity 98%) were purchased from Jingchun Company (Shanghai, China); phloroglucinol (purity 99%) was purchased from Fuchen Company (Tianjin, China); 4-hydroxycinnamic acid (4-OH CA, purity 99%) and 4-chlorocinnamic acid (4-Cl CA, purity 98%) were purchased from Jinsui Company (Shanghai, China). Diazepam, donated by the National Institute on Drug Dependence. Diazepam (DZP), as the positive control, was dissolved in 0.5% sodium carboxyl methyl cellulose (CMC-Na) at a concentration of 0.1 mg/mL. Except for phloroglucinol doses of 0.25, 0.5, and 1 mg/mL, all other natural phenols were prepared at 0.5, 1 and 2 mg/mL with 0.5% CMC-Na. The doses of 4-OH CA and 4-Cl CA were 1, 2, and 4 mg/mL in mouse tests, and 0.7, 1.4 and 2.8 mg/mL in rat tests. The experimental animals received vehicle, diazepam, or one of NSMPs at 0.1 mL/10 g body weight by oral administration. All reagents were freshly prepared on the day of the behavioral tests.

### 2.3. Elevated Plus-Maze Test

The procedure for mice was the same as we previously reported [14]. Briefly, the elevated plus-maze (EPM) apparatus included two open arms (30 × 5 × 1 cm^3^) and two closed arms (30 × 5 × 20 cm) with a central platform (5 × 5 cm). The procedure for rats was performed according to a previously reported protocol with little modification [15]. The test apparatus consisted of two open arms (50 × 10 × 1 cm) and two closed arms (50 × 10 × 40 cm) with a central platform (10 × 10 cm). The arms and platform floors were made of black polypropylene. The walls of the closed arms were made of transparent polypropylene. The floors of the mazes were 50 cm above the room floor. Vehicle, diazepam, or the tested phenols were administered 30 min before the behavioral test. The animals were placed on the central platform facing an open arm with its back toward the experimenter. The movement of the animals on the maze was recorded with a computer-controlled path-tracking system LabState 2.0 (AniLab Software and Instruments Co., Ltd., Ningbo, China). The placing of all four paws (>70% imaged body) on an arm qualified as an entry. The time spent in the open arms (OT) and the entry number (OE) were recorded for 6 min. One entry was defined as the entrance of all four paws into an open arm. The OT and OE were considered to be indicators of the anxiolytic effects. Each animal was tested individually, and the maze was cleaned prior to inserting the next animal. All EPM tests were performed under 140–160 lux between 8:00 a.m. and 1:00 p.m.

### 2.4. Rat Surgery

On the day of surgery, rats were anesthetized using pentobarbital sodium at 50 mg/kg, intraperitoneally (i.p.), and restrained in a stereotactic apparatus (Narishige Co., Tokyo, Japan). The subcutaneous layer of tissue was removed to expose the skull. The implantation position (4.5 mm posterior to bregma, 3.5 mm laterally) for electrode arrays (16 channels) (Plexon Inc., Hong Kong, China) was set according the rat brain atlas drawn by the Paxinos and Watson, and then a hole at this position was drilled in the skull. A microdrive was positioned and the electrode array was lowered through the drilled hole into the CA1 of the left dorsal hippocampus (−2.5 mm relative to the brain surface, CA1 region). The gaps between the electrodes and hole were filled with softened paraffin and the microdrive was secured with dental cement. After completing the surgery, antibiotic penicillin (75,000 U) was i.p. administered for 3–5 days to prevent possible infections. One week after surgery, the EPM test was performed simultaneously with multi-channel in vivo extracellular recordings in the hippocampus CA1, 30 min after the rats received 4-OH CA (14 mg/kg), 4-Cl CA (14 mg/kg), or vehicle.

### 2.5. In Vivo Electrophysiological Recording

Methods were similar to those described previously [16]. In brief, neuronal spikes, filtered at 7–400 kHz and digitized at 40 kHz, were recorded during the entire experimental process using the multichannel acquisition processor system (OmniPlex, Plexon Inc., Dallas, TX, USA.). Spike units were isolated using the Plexon Offline Sorter Software 1.0. The actual recording position was marked by passing a 10-s 20 mA current through two selected electrodes. Figure 3A shows an illustration of the spike unit used for quantification in the in vivo electrophysiological recording. A successful 5-min recording was used to spike counting and frequency calculation. 

### 2.6. Statistical Analysis

Data are expressed as means ± SEM. Behavioral data were analyzed by one-way/two-way analysis of variance (ANOVA) followed by Dunnett’s *t*-test. Chi-square tests were used to analyze electrophysiological data. *p* < 0.05 was considered significant. Statistical software was the SPSS 18.0 (SPSS Inc., Chicago, IL, USA).

## 3. Results

### 3.1. Anxiolytic Effects of NSMPs

The anxiolytic effects of seven NSMPs were tested in the elevated plus maze (EPM) in mice, a classical animal model for anxiety-like behaviors [17]. The typical EPM consists of two parts: open arms and closed arms. The increase of time spent in open arms (OT) and the number of entries into open arms (OE) are taken as anti-anxiety indices. The structures of the tested NSMPs and EPM results are summarized in Table 1. One-way ANOVAs revealed significant differences in OT or OE for each phenol, indicating these phenols were anxiolytic, although their efficiency occurred at different dose levels. Diazepam (DZP, 1 mg/kg) produced significant anxiolytic effects, as indicated by the increase in OT (*p* < 0.05) and OE (*p* < 0.05) compared to the vehicle control. Phloroglucinol altered the OT (5 mg/kg, *p* < 0.05) and the OE (10 mg/kg, *p* < 0.05), and produced a weak but significant anxiolytic effect. Eugenol (5 mg/kg) significantly increased the OT (*p* < 0.05) and OE (*p* < 0.05). Ferulic acid at 5, 10 and 20 mg/kg, and caffeic acid at 10 and 20 mg/kg, considerably prolonged the OT (*p* < 0.01). Protocatechuic aldehyde (10 mg/kg) significantly increased the OT (*p* < 0.05) and OE (*p* < 0.05). Vanillin (5 mg/kg) increased OT (*p* < 0.05) and OE (*p* < 0.05). Thymol (20 mg/kg) significantly affected both the OT (*p* < 0.05) and the OE (*p* < 0.05).

### 3.2. Anxiolytic Differences in 4-OH CA and 4-Cl CA

**Figure 1:**
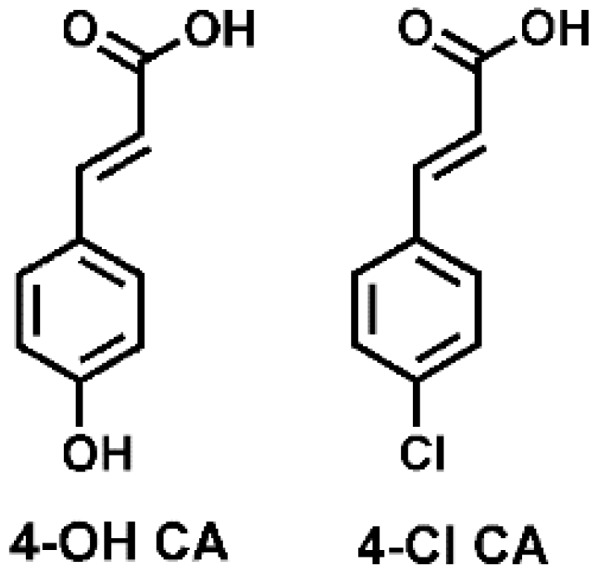
The chemical structures of 4-hydroxycinnamic acid (4-OH CA) and 4-chlorocinnamic acid (4-Cl CA).

**Figure 2:**
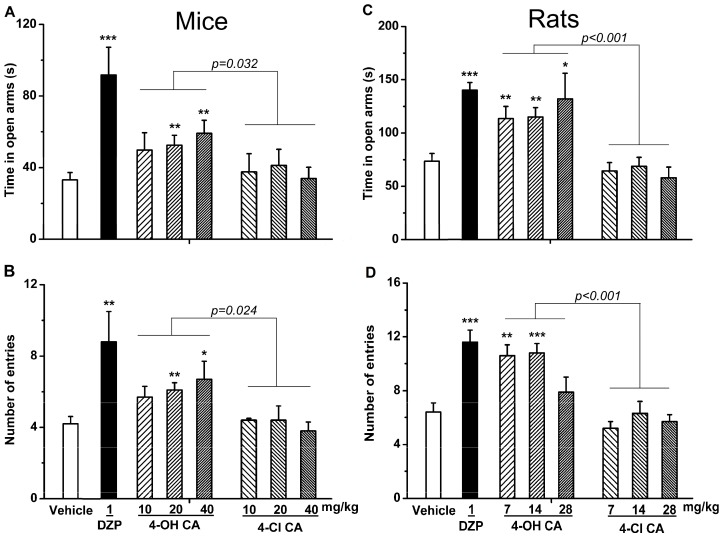
Anxiolytic effects of diazepam (DZP, 1 mg/kg), 4-hydroxycinnamic acid (4-OH CA), and 4-chlorocinnamic acid (4-Cl CA) after intragastric administration in (**A**,**B**) mice and (**C**,**D**) rats. Elevated plus-maze (EPM) results are expressed by (**A**,**C**) the time spent in open arms and (**B**,**D**) the number of open-arm entries. Data are expressed as means ± SEM. * *p* < 0.05, ** *p* < 0.01, *** *p* < 0.001 vs. vehicle control, by Dunnett’s *t*-test.

Summarizing the above results and through reviewing literature on the bioactivities of small-molecule phenols, we focused on the role of the phenol hydroxyl (OH) group in their anxiolytic activities. The essential anxiolytic activity of the phenol moiety in NSMPs was demonstrated by comparing the activity of 4-hydroxycinnamic acid (4-OH CA) and 4-chlorocinnamic acid (4-Cl CA) (Figure 1) in the EPM test in mice and rats at a comparable molar dosage. The experiment data are shown in Figure 2 (A and B for mice; C and D for rats).

In the mouse EPM test, one-way ANOVA revealed significant differences among treatment groups for OT (F(7,79) = 3.744, *p* < 0.01; Figure 2A) and OE (F(7,79) = 5.154, *p* < 0.001; Figure 2B). The post hoc tests (Dunnett’s *t*-test) revealed that mice spent more time in the open arms and went into the open arms more frequently after administration of 4-OH CA (20 and 40 mg/kg), compared to mice in the vehicle group. Conversely, 4-Cl CA had no anxiolytic effects at corresponding doses. One trial of two-way ANOVA between the 4-OH CA and 4-Cl CA groups (treatment × doses) revealed significant drug treatment effects, as shown by the overall significant differences in OT (F(1,16) = 5.504, *p* < 0.05; Figure 2A) and OE (F(1,16) = 6.192, *p* < 0.05; Figure 2B). No significant differences existed in the effects of the doses.

To verify the above experimental results, the anxiolytic effects of 4-OH CA and 4-Cl CA were assayed using the rat’s EPM (Figure 2C,D). The results revealed that 4-OH CA significantly increased OT (F(7,64) = 7.688, *p* < 0.001; Figure 2C) and OE (F(7,64) = 8.976, *p* < 0.001; Figure 2D), indicating significant differences between the groups. For rats, the post hoc tests revealed that OT and OE in the diazepam (1 mg/kg) and 4-OH CA (7 and 14 mg/kg) groups were significantly higher than those in the vehicle group. Two-way ANOVA the between 4-OH CA and 4-Cl CA groups (treatment × doses) revealed significant drug treatment effects, as shown by the overall significant differences in OT (F(1,16) = 24.326, *p* < 0.001; Figure 2C) and OE (F(1,16) = 29.869, *p* < 0.001; Figure 2D). No significant differences were observed in the dose effects. The results in rats showed that diazepam and 4-OH CA were anxiolytic in the EPM test, consistent with the results from the mouse experiments.

### 3.3. Electrophysiological Differences in 4-OH CA and 4-Cl CA

**Figure 3:**
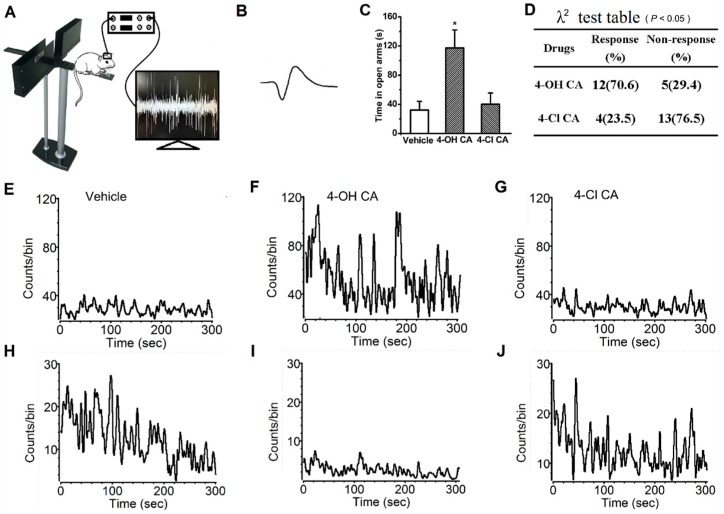
4-OH CA and 4-Cl CA differently affect the neuronal spikes in hippocampus of rats. (**A**) The in vivo neuronal spike recording in rats; (**B**) the typical neuronal spike quantified here; (**C**) 4-OH CA (14 mg/kg) is anxiolytic, but not 4-Cl CA (14 mg/kg); (**D**) comparison of the effect of 4-OH CA and 4-Cl CA on specific neuronal spikes in five-minute time intervals 30 min after drug administration; (**E**–**G**) one neuron shows enhanced spike responses to 4-OH CA, but not 4-Cl CA; and (**H**–**J**) one neuron shows inhibited responses to 4-OH CA, but not 4-Cl CA.

The differences in 4-OH CA and 4-Cl CA in neural activity were demonstrated by their effects on neuronal spikes in the rat hippocampus. After the rats recovered from the implantation of the electrode array into the hippocampal A1 region, the anxiety-related behaviors and spikes of the hippocampal neurons were simultaneously monitored (Figure 3A). As shown in Figure 3C, 4-OH CA significantly increased OT compared to the vehicle and 4-Cl CA (14 mg/kg) groups, 30 min after intragastric administration (F(2,15) = 9.463, *p* < 0.01), indicating the implantation surgery did not affect the brain structures (hippocampal A1 region) in response to 4-OH CA. From the four rats under in vivo spike recording, 17 neurons with typical spikes (Figure 3B) were selected to assay their responses to 4-OH CA (14 mg/kg) and 4-Cl CA (14 mg/kg). The responses were defined as an increase in spike frequency (at least double those of the vehicle group) or a decrease in spike frequency (50% of vehicle or lower). The data in Figure 3D are the quantitative judgment of response or non-response, showing that the difference between groups (4-OH CA and 4-Cl CA) was significant (*p* < 0.01, λ^2^-test). The data in Figure 3E–G are the spike responses from one hippocampal neuron after administration of vehicle, 4-OH CA, or 4-Cl CA, respectively, demonstrating 4-OH CA increased spike responses, but 4-Cl CA did not. The data in Figure 3G,H are the spike responses from another hippocampal neuron after administration of vehicle, 4-OH CA, or 4-Cl CA, respectively, demonstrating 4-OH CA decreased spike responses, but 4-Cl CA did not.

## 4. Discussion

The EPM is a classical model to assess anxiety-like behaviors in mice [17]. Using this test, the anxiolytic activity of phloroglucinol, eugenol, protocatechuic aldehyde, vanillin, ferulic acid, and thymol, were revealed for the first time, demonstrated by significant increases in the OT or OE compared to the vehicle control. The anxiolytic activities of caffeic acid [18] and 4-hydroxycinnamic acid [19] were consistent with previously-reported studies. Furthermore, the dominative role of the phenol moiety was proven by the substitution of an OH group in 4-hydroxycinnamic acid by chlorine atom (changing to 4-chlorocinnamic acid) leading to the loss of anxiolytic activity, which was observed at comparable dosages. In addition, a significant difference between 4-OH CA and 4-Cl CA was found in their effects on neuronal spikes in the rat hippocampal CA1 region, indicating that influencing the neuronal spikes in CA1 might be one of the anxiolytic pathways of 4-OH CA.

The above behavioral findings directed us to perform a systematic literature review. NSMP is specifically defined here as a natural compound whose molecular weight is less than 350 Dalton, and contains only carbon (C), hydrogen (H), oxygen (O), and at least one OH in the structure. Using SciFinder Scholar, we discovered 54 anxiolytic NSMPs as of June 2016 (a full list of NSMPs is not shown in this paper; part of them can be found be one of our review [20]). The anxiolytic activity of a single phenol (carbolic acid) has not been documented, perhaps due to its strong toxicity [21]. However, the reported anxiolytic NSMPs include those phenols whose molecular weight is as low as possible, such as orcinol [14], paeonol [6], gallic acid [22], carvacrol [8], and those in this study (Table 1). Phloroglucinol contains three phenolic hydroxyl groups and has no other substitutions on the phenyl skeleton. The structural diversity, other than phenol moiety of these NSMPs, demonstrated the key role of the phenol group. As shown in Table 1, and as with the 54 anxiolytic NSMPs identified from SciFinder database, these phenols have a narrow therapeutic window (several to several tens of mg/kg), indicating their efficacy may be strictly controlled by the chemical properties of phenol-OH. In other words, the moiety of phenol may act as a carrier of pharmacological signals. Another interesting finding suggests those phenols with relatively higher molecular weight, like kaempferol and quercetin, should be metabolized to smaller phenols, like (*p*-hydroxyphenyl acetic acid and 3,4-dihydroxyphenylacetic acid, for the expression of their anxiolytic effects [9]. Thus far, our hypothesis that NSMPs are anxiolytic is reasonable. Additionally, we hypothesize that small phenols are anxiolytic, and not only in natural products, because some synthesized small-molecule phenols, such as propofol and isobutylparaben, are also anxiolytic [23,24].

To determine the anxiolytic activities of NSMPs, their overlaps of pharmacological mechanisms should be checked. Several clarified mechanisms contribute to the effects of clinical anxiolytic drugs, such as their actions on reversing the imbalance of neurotransmitters, neuroendocrine dysfunction, or dysimmuneneuropathy [25]. In our previous research, we demonstrated that orcinol can penetrate the blood–brain barrier [14], indicating the possibility that NSMPs act on the central nervous system. For the anxiolytic mechanism, numerous references report that NSMPs have effects on neurotransmitters and responding receptors. GABA_A_ receptor-mediated signaling may be the most probably NSMP target. The anxiolytic effects of effusol [26], honokiol [27], sinapic acid [28,29], obovatol [30], 4-hyroxybenzaldehyde [31], 6-hydroxyflavone [32], chrysin [33], apigenin [34], wogonin [35], *p*-coumaricacid [19], ellagic acid [36], and baicalein [37,38] were proven to be related to the GABA_A_ receptor or its signaling cascade. The GABA_A_ receptor may be one of the pharmacological targets of NMSPs. In addition to GABA signaling, other monoamine neurotransmitters may be involved. As anxiolytics, myricetin [39], epicatechin [40], 4-hyroxybenzyl alcohol [31], gallic acid [41], and cannabidiol [42], were reported to act through the serotonin pathway. Caffeic acid was found to be related to the adrenergic receptor [18], and danshensu worked through dopamine signaling [10]. Until now, no papers on NSMPs have been published revealing the anxiolytic mechanism beyond the monoamine neurotransmitter. Numerous brain regions have been identified associated with the expression of anxiety-like behaviors, including the hippocampus, amygdala, prefrontal cortex, and so on [43]. Judging from spike frequency, hippocampal neurons were inhibited or enhanced, or not affected after administration of 4-OH CA. From limitations of this study, we cannot draw a conclusion on which kind of response is related the anxiolytic effects of 4-OH CA. Results in Figure 3 show that hippocampus CA1 region may be involved in the anxiolytic mechanism of 4-OH CA, and possibly for other NSMPs. However, because of the response diversity of neuronal spikes and multiple hierarchical brain structures involved in anxiety modulation, it is difficult to say that the molecular targets (GABA_A_ receptor and/or others) are located in hippocampal CA1 region.

**Figure 4:**
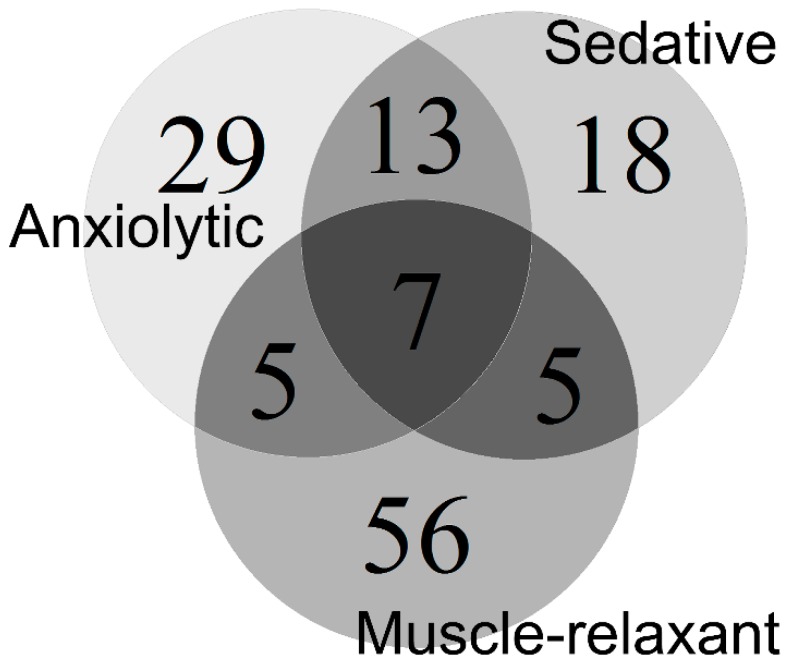
Bioactivity overlap of 133 NSMPs.

Other bioactivities of NSMPs also support the idea of considering NSMPs as a whole. The typical anxiolytic benzodiazepines, such as diazepam, show sedative and muscle-relaxant effects at relatively high doses [44,45]. By searching the SciFinder database as of June 2016, we chose 43 NSMPs with sedative activities, and 73 NSMPs with muscle-relaxant/spasmolytic activities (Figure 4). A full list of these NSMPs is not provided in this paper. Dehydroeffusol [11,46], apigenin [34,47], chrysin [33,48], kaempferol [9,49,50], luteolin [51,52,53], quercetin [9,54,55], and ellagic acid [36,56,57] are anxiolytic, sedatives, and muscle-relaxants, similar to the pharmacological profile of diazepam. NSMPs have other common bioactive properties, as mentioned previously. Although likely to be disputed, we would like to introduce a new word, “phenolism”, to pharmacologically cover these properties, including, but not limited to, anti-oxidation, anti-microorganism, anti-spasm, sedation, neuroprotection, and anti-anxiety activities. More cycles are provided in Figure 4, and maximum overlap is expected.

## 5. Conclusions

NSMPs extensively exist in herbal medicines, vegetables, fruits, or plant-source food. However, systemic studies are lacking on the effects of NSMPs on human anxiety when receiving a sufficient dose. Our hypothesis, although expecting more supportive evidence, may support the reasonable use of herbal medicines and new anxiolytic drug development. For example, we could attempt to discover new phenol-rich plants or their extractions to treat anxiety, or to control the everyday intake of NSMPs in food to modulate mood. Furthermore, our hypothesis may be helpful for understanding the pharmacological mechanisms of herbal medicine traditionally used to treat anxiety.

## Figures and Tables

**Table 1:** The chemical structures, names, and anxiolytic activities of seven natural small-molecule phenols (NSMPs).

Structure and Name	Dose (mg/kg)	Anxiolytic Activity		ANOVA
		OT	OE	
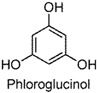	Vehicle	49.6 ± 6.5	4.8 ± 0.5	OT: F(4,45) = 3.245, *p* = 0.020 OE: F(4,45) = 2.730, *p* = 0.041
	DZP	116.1 ± 25.8 *	11.1 ± 3.0	
	2.5	67.2 ± 9.2	8.4 ± 1.4 *	
	5	74.8 ± 7.4 *	6.5 ± 0.7	
	10	69.9 ± 9.1	7.5 ± 1.2 *	
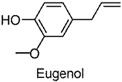	Vehicle	31.0 ± 5.8	4.0 ± 0.5	OT: F(3,36) = 3.261, *p* = 0.033 OE: F(3,36) = 3.434, *p* = 0.027
	5	53.3 ± 8.7 *	6.1 ± 0.8 *	
	10	39.6 ± 7.1	4.5 ± 0.9	
	20	41.9 ± 9.1	4.3 ± 0.7	
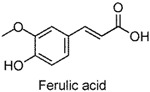	Vehicle	63.8 ± 7.5	8.1 ± 0.7	OT: F(3,36) = 4.818, *p* = 0.006 OE: F(3,36) = 1.099, *p* = 0.362
	5	103.2 ± 9.8 **	10.2 ± 0.8	
	10	93.5 ± 8.7 *	9.5 ± 0.9	
	20	106.1 ± 9.1 **	9.1 ± 0.9	
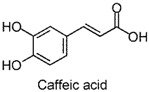	Vehicle	63.8 ± 7.5	8.1 ± 0.7	OT: F(3,36) = 5.932, *p* = 0.020 OE: F(3,36) = 1.320, *p* = 0.283
	5	76.6 ± 9.6	8.5 ± 1.3	
	10	101.4 ± 4.2 ***	9.1 ± 0.6	
	20	95.5 ± 6.0 **	10.7 ± 1.1	
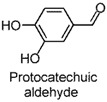	Vehicle	34.7 ± 6.4	3.7 ± 0.4	OT: F(3,36) = 3.349, *p* = 0.030 OE: F(3,36) = 2.910, *p* = 0.048
	5	47.8 ± 8.3	4.3 ± 0.7	
	10	63.4 ± 5.9 **	5.7 ± 0.6 *	
	20	49.3 ± 7.6	5.2 ± 0.7	
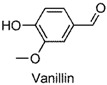	Vehicle	34.7 ± 6.4	3.7 ± 0.4	OT: F(3,36) = 3.790, *p* = 0.018 OE: F(3,36) = 3.163, *p* = 0.036
	5	52.9 ± 4.0 *	5.4 ± 0.6 *	
	10	41.9 ± 5.8	4.0 ± 0.7	
	20	41.4 ± 8.4	4.6 ± 0.7	
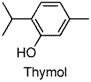	Vehicle	31.0 ± 5.8	4.0 ± 0.5	OT: F(3,36) = 3.261, *p* = 0.033 OE: F(3,36) = 3.188, *p* = 0.035
	5	49.3 ± 7.2	5.9 ± 0.9	
	10	38.8 ± 8.4	4.0 ± 0.6	
	20	59.1 ± 5.3 **	5.9 ± 0.7 *	

Note: Data are expressed as means ± SEM (n = 10 for each group). DZP = diazepam 1 mg/kg, as positive control; OT = Time spent in the open arms (s); OE = Entry number into the open arms. * *p* < 0.05, ** *p* < 0.01, *** *p* < 0.001 vs. vehicle as revealed by Dunnett’s *t*-test. Eugenol and thymol were tested during the same time course so that they shared the same vehicle control, as were ferulic acid and caffeic acid.
